# Susceptibility to emotional contagion for negative emotions improves detection of smile authenticity

**DOI:** 10.3389/fnhum.2013.00006

**Published:** 2013-03-18

**Authors:** Valeria Manera, Elisa Grandi, Livia Colle

**Affiliations:** ^1^Center for Cognitive Science, Department of Psychology, University of TurinTorino, Italy; ^2^Stanford Psychophysiology Laboratory, Department of Psychology, Stanford UniversityStanford, CA, USA

**Keywords:** smile authenticity, emotional contagion, simulation models, individual differences, positive and negative emotions

## Abstract

A smile is a context-dependent emotional expression. A smiling face can signal the experience of enjoyable emotions, but people can also smile to convince another person that enjoyment is occurring when it is not. For this reason, the ability to discriminate between felt and faked enjoyment expressions is a crucial social skill. Despite its importance, adults show remarkable individual variation in this ability. Revealing the factors responsible for these huge individual differences is a key challenge in this domain. Here we investigated, on a large sample of participants, whether individual differences in smile authenticity recognition are accounted for by differences in the predisposition to experience other people's emotions, i.e., by susceptibility to emotional contagion. Results showed that susceptibility to emotional contagion for negative emotions increased smile authenticity detection, while susceptibility to emotional contagion for positive emotions worsened detection performance, because it leaded to categorize most of the faked smiles as sincere. These findings suggest that susceptibility to emotional contagion plays a key role in complex emotion recognition, and point out the importance of analyzing the tendency to experience other people's positive and negative emotions as separate abilities.

## Introduction

A smile is a context-dependent emotional expression. A smiling face does not always signal the experience of enjoyable emotions: sometimes people smile to convince another person that enjoyment is occurring when it is not. This can be done for many different reasons, for example to hide, moderate, or justify something negative (e.g., a feeling of superiority or contempt, a manipulation, social embarrassment, or an inappropriate affect), or simply to coordinate conversation. The ability to discriminate between felt and faked enjoyment expressions is critical to effective social interaction, and to cope with the complexity of the human social world: recognizing a faked smile can prevent people from being deceived, from being inappropriate in a formal social situation, or from starting a potentially frustrating collaboration with a person with no cooperative intents (see Miles, 2009; Johnston et al., 2010). Despite recognizing the authenticity of smiles plays such a crucial role in dealing with everyday social interactions, there are striking individual differences in this ability: whereas some people are very good at distinguishing felt and simulated enjoyment expressions, others appear to lack this ability more or less completely (Frank et al., 1993; Gosselin et al., 2002; Del Giudice and Colle, 2007; McLellan et al., 2009; see Ekman, 2003 for a review). Which are the factors responsible for this remarkable individual variation? Despite unrevealing these factors is a key challenge in this domain, very few studies so far have directly addressed this question (see Manera et al., 2011).

A good way to derive workable hypotheses on the factors responsible for individual differences is starting from the mechanisms involved in smile authenticity detection. How can we distinguish felt from faked smiles by simply looking at other people's face? To date, research on smile recognition has almost exclusively focused on perceptual factors, and has shown that there are substantial perceptual differences between different types of smiles. Smiles judged as genuine involve the activation of specific muscle regions (e.g., the external strand of the *Obircularis Oculi* muscle, producing the narrowing of the eye aperture, and the appearance of *crow's feet* on the external side of the eye—also known as Duchenne marker, Frank et al., 1993), display smooth and more regular facial movements, and are longer in onset, apex and offset durations (Krumhuber and Manstead, 2009) compared to smiles that are judged as less genuine. Moreover, it has been shown that these dynamic and morphological cues are consistently used by observers in order to rate smile authenticity (Frank et al., 1993; Del Giudice and Colle, 2007; Miles and Johnston, 2007; Krumhuber and Manstead, 2009). However, we have recently shown that perceptual factors—such as the attention devoted to the eye region—do not seem to account for the striking variation in smile recognition accuracy across individuals (Manera et al., 2011). This suggests that other mechanisms are likely to be involved in smile authenticity detection.

Recently, it has been proposed that smile recognition relies on *embodied simulation* processes (Niedenthal et al., 2010). Embodied simulation models advance that observers automatically mimic other people's facial expressions, experience those emotions themselves, and consequently attribute them to the other person. Thus, in this account, smile recognition is based on first-person emotional experience (see Goldman and Sripada, 2005). This proposal allows to formulate new hypotheses concerning possible sources of individual variation in smile authenticity recognition. An intriguing hypothesis which—to our knowledge—has never been explored, is that the ability to distinguish between sincere and faked enjoyment expressions is affected by the predisposition to experience others' emotions, i.e., by susceptibility to emotional contagion. In the present paper we focused on this question, and we asked whether individual differences in smile authenticity recognition are accounted for by differences in the susceptibility to emotional contagion. In the following paragraphs we will first briefly review the embodied simulation account of smile recognition proposed by Niedenthal et al. (2010). We will then focus on emotional contagion, and we will finally introduce the present study.

### Simulation models of smile recognition

The Simulation of Smile Model (Niedenthal et al., 2010) advanced the hypothesis that we distinguish different categories of smile based on embodied simulation processes. Embodied simulation models posit that the perception of a facial expression automatically triggers the activation of the facial configuration associated with the observed emotion (*mimicry*), and this, in turn, induces in the observers the physiological activations and the subjective experience of the very same emotion (*emotional contagion)*. The experienced emotion is then attributed to the other person (Adolphs, 2006; see Goldman and Sripada, 2005 for a review of the different existing versions of face-based embodied simulation models). Even if not conclusive, there is consistent evidence in favor of simulation accounts of emotion recognition. We know that observers rapidly and automatically mimic other people's emotional expressions (Hess et al., 1998; Dimberg et al., 2000; Lishner et al., 2008), and that mimicry can aid emotion recognition. For instance, mimicking other's expressions results in faster emotion categorization (Stel and van Knippenberg, 2008), while blocking facial mimicry can impair emotion recognition (Oberman et al., 2007; Ponari et al., 2012; but also see Hess and Blairy, 2001). Furthermore, the observation of other people's emotional expressions is able to induce in the perceiver the experience of the corresponding emotions, i.e., emotional contagion (Hsee et al., 1992; Strayer, 1993; Schneider et al., 1994; Blairy et al., 1999; Wild et al., 2001; Lishner et al., 2008).

In a recent study, Maringer et al. (2011) provided preliminary evidence in favor of the SIMS model, demonstrating that mimicry can aid in smile authenticity recognition. Two groups of participants were presented with smiles with different dynamic qualities known to be associated with “true” or “false” smiles, and were asked to rate them on a scale of genuineness. During the task, half of the participants in each group were able to freely mimic the smiles, and the remaining half held a pencil in their mouths so as to block facial mimicry. In the mimicry condition, participants who saw true smiles rated them as more genuine compared to participants who saw false smiles. In the mimicry-blocked condition, no difference in the authenticity ratings was found between participants who saw true and false smiles. Although this study employed a between-subject design (not taking into account the striking individual differences in both smile genuineness recognition and tendency to mimic other people's emotions and behaviors), these results support the idea that simulation processes play a role in smile authenticity recognition. If this is the case, also emotional contagion should play a role in smile recognition.

### Emotional contagion

Emotional contagion refers to the human tendency to automatically mimic and synchronize facial expressions, vocalizations, postures and movements with those of another person, and consequently converge emotionally with them (Hatfield et al., 1994). This basic form of empathy (Preston and de Waal, 2002; Singer, 2006) allows us to share the emotions of others directly, without any conscious effort and any form of cognitive mediation. Through emotional contagion we feel others' emotions directly in our body, as if they were our own emotions. Emotional contagion is not considered a single and undifferentiated mechanism; rather—since different emotions are characterized by distinct facial expressions, psycho-physiological patterns, and brain activations (LeDoux, 2000; Dalgleish, 2004)—it may be modulated by the specific emotional content of the observed stimuli (see Goldman and Sripada, 2005). In particular, emotional contagion for positive emotions and emotional contagion for negative emotions may be conceived as partially distinct mechanisms, as positive and negative emotions show differential facial and physiological activations (Schwartz et al., 1979; Davidson et al., 1990), and engage non-completely overlapping neural circuits (Adolphs et al., 1996). It has been proposed that the neural substrate responsible for emotional contagion is represented by the Mirror Neuron System for emotions, including the insula and the anterior mesial frontal cortex. This system is active both when we feel a specific emotion, and when we see another person's emotional expressions (see Keysers and Gazzola, 2009, and Rizzolatti and Sinigaglia, 2010 for reviews).

The SIMS model advanced the prediction that emotional contagion has an impact on smile authenticity recognition. Although it has never been empirically tested, this hypothesis is very plausible. There is evidence that felt (Duchenne) smiles are associated with the experience and physiological activations of positive emotions, while faked non-Duchenne smiles are associated with the experience and physiological activation of negative emotions (Davidson et al., 1990; Ekman et al., 1990; Soussignan, 2002). For this reason, it is possible to expect that felt and faked smiles recruit different components of emotional contagion.

In the present study we did not address directly this hypothesis on emotional contagion. As we were interested in individual differences, we focused instead on *susceptibility to emotional contagion*, i.e., a trait measure strongly associated to online measures of emotional contagion.

#### Individual differences in the susceptibility to emotional contagion: the present study

Susceptibility to emotional contagion, i.e., the predisposition to converge emotionally with other people, shows remarkable variations across individuals. While some people show a strong tendency to experience others' emotions, some others seem to be scarcely affected by the observation of others' emotional states, as testified by huge individual differences in self-reported emotional contagion (Doherty, 1997; Sonnby-Borgstrom, 2002). The individual predisposition to experience emotional contagion can be reliably measured through a number of self-report questionnaires, such as the Personal Distress subscale of the Interpersonal Reactivity Index (Davis, 1983), and the Emotional Contagion Scale (ECS) (Doherty, 1997). People scoring higher on these trait measures are more prone to experience other people's emotions, and mimic others' emotional expressions more consistently compared to people with a low susceptibility to emotional contagion (e.g., Hietanen et al., 1998; Blairy et al., 1999). Furthermore, when observing other people's emotional expressions, persons scoring higher in emotional contagion show stronger activation of brain areas in the mirror neuron system for emotions, such as the insula, inferior-parietal junction, and anterior cingulated cortex (Lawrence et al., 2006; Lamm et al., 2007; Pfeifer et al., 2007).

If simulation processes and emotional contagion are involved in smile authenticity recognition, as the SIMS model advanced, individual differences in the susceptibility to emotional contagion may account for individual differences in the ability to distinguish sincere and faked enjoyment expressions. Here we tested this hypothesis on a large sample of participants. Smile authenticity detection was assessed by means of the Smile Picture Set (SPS), a validated FACS-based task including sincere Duchenne smiles and simulated non-Duchenne smiles (Del Giudice and Colle, 2007). Susceptibility to emotional contagion was measured through the ECS (Doherty, 1997), a validated self-report questionnaire specifically designed to measure individual differences in susceptibility to emotional contagion. This self-report measure has been widely employed in a number of domains, and, to our knowledge, it is the only validated instrument tapping both positive and negative emotions. As discussed in the previous paragraphs, sincere Duchenne smiles are associated with the experience and physiological activations of positive emotions, while faked non-Duchenne smiles are associated with the experience and physiological activation of negative emotions (Davidson et al., 1990; Ekman et al., 1990; Soussignan, 2002). For this reason, it is plausible to expect that susceptibility to emotional contagion for positive and negative emotions play a different role in predicting individual differences in smile authenticity recognition.

## Materials and methods

### Participants

One hundred and eight undergraduate students (58 females, 50 males) from the University of Torino volunteered to participate in the study. The average age was 22 years (range = 18–34 years). All participants had normal or corrected-to-normal vision and were naive with respect to the purpose of the experiment. This research was approved by the local Ethical Committee in line with the Declaration of Helsinki.

### Materials and procedure

Participants were administered a FACS-based task evaluating the ability to detect smile authenticity from facial expressions (the *Smiles Picture Set*, Del Giudice and Colle, 2007), and a self-report questionnaire evaluating the tendency to experience emotional contagion. The tasks were administered individually in a randomized order, and took about 20 min to complete.

#### Smile recognition

Smile recognition was assessed through the SPS (Del Giudice and Colle, 2007), a validated FACS-based task consisting of 25 color pictures of an actor's face displaying smiles of variable intensity, either with closed lips (AU12) or bared teeth (AU12 + AU25). The set contains 11 Duchenne smiles with AU6 activation and 14 non-Duchenne smiles (seven with AU7 activation and seven with a neutral eye region). Complete FACS codings of the SPS pictures can be found in Del Giudice and Colle (2007). Previous research has demonstrated that the Duchenne smiles included in this set are rated as significantly more genuine compared to the non-Duchenne smiles (Del Giudice and Colle, 2007; Manera et al., 2011).

Stimuli were displayed on a 21″ monitor by means of Presentation 9.3 software (Neurobehavioral Systems), at a viewing distance of 70 cm. Pictures had a resolution of 1024 by 768 pixels, and subtended a vertical visual angle of 15° and a horizontal angle of 22°. The task started with a preliminary phase, in which four pictures of the same actor performing different expressions (anger, sadness, surprise, and disgust) were presented, each preceded by the picture of the actor's neutral face. This phase gave participants the opportunity to get accustomed to the actor's face. The 25 items were then shown in one of two randomized orders; each item had a duration of 3 s and started with a neutral face followed by the smiling face. After each stimulus presentation, participants were asked to decide whether the actor was really happy, or was pretending to be happy. Responses were given by pressing one of two keys on a keyboard. In order to reduce guessing, participants were allowed to abstain from responding if they were really unsure about their answer.

#### Susceptibility to emotional contagion

The individual tendency to experience emotional contagion was measured through the ECS (Doherty, 1997), a widely used self-report questionnaire. ECS is a 15-item scale that separately evaluates the susceptibility to emotional contagion for positive emotions (happiness and love, six items) and negative emotions (fear, anger and sadness, nine items). Examples are: “If someone I'm talking with begins to cry, I get teary-eyed” and “When someone smiles warmly at me, I smile back and feel warm inside.” Participants were asked to rate their degree of agreement with each item on a 5-point scale, from “never” to “always.”

### Data analysis

Performance in the smile recognition task was assessed by means of the proportion of correct responses (accuracy) and the Signal Detection Theory parameter *d*′ (sensitivity). To calculate accuracy, each response was coded as correct (1 point) or incorrect (0 points). A correct response was scored for each “really happy” answer to Duchenne smile items, and for each “pretending to be happy” answer to non-Duchenne smile items. “Don't know” answers were awarded 0 points. As in yes-no tasks the proportion of correct responses represents a biased measure (i.e., it does not consider systematic errors in performance), we also extracted Signal Detection Theory parameters. “Don't know” answers were coded as invalid trials, and were excluded from the analysis. The proportion of hits (“really happy” answer to valid Duchenne smile trials) and false alarms (“really happy” answer to valid non-Duchenne smile trials) were used to calculate the location of the criterion *c* (i.e., the general tendency to respond “really happy” or “pretending to be happy”; e.g., a value of zero indicates no bias) and the *d*′, an unbiased sensitivity index-independent of the criterion the participant is adopting (e.g., a value of zero indicates an inability to discriminate Duchenne smile trials from non-Duchenne smile trials, whereas larger values indicate a correspondingly greater ability to discriminate between them). Hits and false alarm proportions of zero were replaced with 0.5/N, and proportions of 1 were replaced with (N-0.5)/N (where N is the number of valid Duchenne smile and non-Duchenne smile trials for each participant).

To explore the effects of gender, accuracy, *d*′, *c* and emotional contagion scores were submitted to separate ANOVAs with participant's gender as within-subject factor. Pearson correlations between the two emotional contagion subscales were performed. To explore the impact of susceptibility to emotional contagion on smile recognition, accuracy, *d*′ and error type (false alarm rate and miss rate) were submitted to separate linear regressions, with the two emotional contagion scales (ECS_pos and ECS_neg) as regressors.

## Results

Consistent with previous studies, major individual differences in smile authenticity detection were found. The proportion of correct responses in the SPS ranged from 0.28 to 1.00, with a mean of 0.68 (*SD* = 0.13). “Don't know” responses were less than 10%. *d*′ ranged from −0.53 to 3.53 (*M* = 1.46, *SD* = 0.30). Criterion *c* ranged from −0.76 to 1.82 (*M* = 0.10, *SD* = 0.48), and was significantly greater than 0 [*t*_(107)_ = 2.13, *p* = 0.036], thus indicating that participants, as a group, adopted a conservative criterion (i.e., had a slight tendency to respond “pretending to be happy”). No gender differences in accuracy, *d*′ and *c* were found [*F*_(1, 107)_ ranging from 0.48 to 1.47, *p*_*s*_ ranging from 0.23 to 0.49].

ECS_pos scores ranged from 13 to 30 (*M* = 23.6, *SD* = 3.5), and ECS_neg scores ranged from 12 to 37 (*M* = 24.7, *SD* = 5.3). Consistent with previous findings (e.g., Doherty, 1997), significant gender differences in susceptibility to emotional contagion were found, with females scoring higher than males [ECS_pos: *F*_(1, 107)_ = 7.3, *p* = 0.01; ECS_neg: *F*_(1, 107)_ = 13.5, *p* < 0.001]. A positive correlation between ECS_neg and ECS_pos was found [*r*_(106)_ = 0.51, *p* < 0.001].

Results of regression analysis for accuracy, *d*′, false positive and false negative error rate are reported in Table 1.

**Table 1:** **Linear regression analyses of how emotional contagion scales predict smile authenticity recognition (accuracy and *d*′) and error types (false alarm rate and miss rate)**.

**Emotional contagion**	***B***	**Beta**	***T***	***P***
**DEPENDENT VARIABLE: *ACCURACY* - *F* TEST: *P*= 0.026; *R*^2^ = 0.07**				
ECS_pos	−0.24	−0.25	−2.31	0.023
ECS_neg	0.17	0.27	2.45	0.016
**DEPENDENT VARIABLE: *d*′ - *F* TEST: *P* = 0.003; *R*^2^ = 0.10**				
ECS_pos	−0.07	−0.30	−2.78	0.006
ECS_neg	0.05	0.34	3.20	0.002
**DEPENDENT VARIABLE: *FALSE ALARM RATE* - *F* TEST: *P* = 0.003; *R*^2^ = 0.09**				
ECS_pos	0.02	0.35	3.22	0.002
ECS_neg	−0.01	−0.30	−2.78	0.006
**DEPENDENT VARIABLE: *MISS RATE* - *F* TEST: *P* = 0.348; *R*^2^ = 0.001**				
ECS_pos	0.005	0.08	0.67	0.502
ECS_neg	−0.007	−0.16	−1.46	0.148

Emotional contagion was a significant predictor of performance for both accuracy [*F*_(2, 107)_ = 3.77, *p* = 0.026, *R*^2^ = 0.07] and *d*′ [*F*_(2, 107)_ = 6.02, *p* = 0.003, *R*^2^ = 0.10], suggesting that around 10% of the individual differences in smile authenticity recognition found in the present study were explained by differences in the susceptibility to emotional contagion. Interestingly, emotional contagion for negative emotions (ECS_neg) was found to be a positive predictor of participants' performance (accuracy: *p* = 0.016; *d*′, *p* = 0.002): the higher the scores on emotional contagion for negative emotions, the higher the performance in the smile detection task. On the contrary, emotional contagion for positive emotions (ECS_pos) was negatively correlated with accuracy and *d*′ (accuracy: *p* = 0.023; *d*′, *p* = 0.006), thus suggesting that the higher the score on emotional contagion for positive emotions, the lower the performance in the smile detection task. Regressions between error-type (miss and false alarm rate) and emotional contagion scores suggest a reason for this pattern of results: people scoring higher on ECS_pos performed worse in smile recognition because they made more false alarms (*p* = 0.002), i.e., they had a tendency to rate non-Duchenne smiles as sincere. The reverse pattern was found for ECS_neg, indicating that people scoring higher on ECS_pos made fewer false alarms (*p* = 0.006). No significant effect of emotional contagion on “miss” errors was found.

## Discussion and future research directions

Emotional contagion—the tendency to unconsciously mimic others' emotional expressions and, consequently, converge emotionally with them (Hatfield et al., 1994)—is a core aspect of human social functioning. People more susceptible to emotional contagion are more sensitive to others, have a higher self-esteem, and are more empathic compared to people who are less affected by others' emotions (Doherty, 1997). Hatfield et al. (1994) proposed that people who are more susceptible to emotional contagion are also better at reading others' emotional expressions. However, evidence in this respect is still sparse and contradictory (Riggio et al., 1989; Levenson and Ruef, 1992; Blairy et al., 1999). Here we showed for the first time that susceptibility to emotional contagion is related to the ability to discriminate sincere and faked enjoyment expressions: individual differences in emotional contagion accounted for around 10% of individual variation in smile authenticity recognition. Given that emotional contagion was assessed indirectly through self-reports, this percentage is notably high. Indeed, using the same smile recognition task, Manera et al. (2011) showed that perceptual factors (such as the attention devoted to the eye-region), which are well-known to play a role in smile recognition, explained less than 5% of inter-individual variation.

Interestingly, we demonstrated that susceptibility to emotional contagion for positive and negative emotions play a different, opposite role in smile authenticity detection. We found that participants with higher susceptibility to emotional contagion for negative emotions performed better in smile authenticity detection, and they made fewer “false positive” mistakes, that is, they rarely rated faked stimuli as sincere enjoyment expressions. On the contrary, participants with higher susceptibility to emotional contagion for positive emotions showed a reduced sensitivity in detecting emotion authenticity, specifically because they had a tendency to rate non-Duchenne smiles as sincere.

These findings support the hypothesis that susceptibility to emotional contagion is strongly influenced by its emotional content. Faked, non-Duchenne smiles are associated with the experience and physiological activation of negative emotions (Davidson et al., 1990; Ekman et al., 1990; Soussignan, 2002). People with high susceptibility to emotional contagion for negative emotions may be especially sensitive to cues of negative emotions (e.g., the absence of the Duchenne marker in the eye region of a smiling person), and specifically resonate with them, thus experiencing negative emotions. This focus on negative emotions may thus enhance their ability to detect faked smiles (which, in a task where they are asked to rate smile authenticity, translate into a better discrimination performance). Converging with this interpretation, it was found that socially rejected individuals, who have a tendency to focus on negative emotions such as sadness and anger, show an enhanced ability to determine whether an enjoyment facial expression is genuine or deceptive (Bernstein et al., 2008). On the contrary, people inclined to resonate with others' positive emotions may focus especially on the expressive cues signaling positive feelings (e.g., a big smile), experience positive feelings, and, as a consequence, they may overestimate others' happiness, and be easily deceived by subtly faked facial expressions. According to this interpretation, it has been found that older people—who show general biases in focusing on positive information (see Carstensen et al., 2003) have a greater bias toward reporting that any smiling individual is feeling happy, independently of whether he/she is displaying enjoyment or non-enjoyment smiles (Slessor et al., 2010). In order to test whether this interpretation is correct, it would be interesting to examine whether susceptibility to emotional contagion for positive and negative emotions predicts an increased attentional focus on positive and negative emotional cues, respectively, and/or whether this is related to online measures of mimicry and emotional contagion. Further studies would be needed in order to test these predictions. In particular, it would be important to directly investigate whether susceptibility to emotional contagion and online measures of emotional contagion (e.g., autonomic activity) provide converging results concerning the way they affect smile authenticity recognition.

Even if we focused on susceptibility to emotional contagion, which is a trait measures, we believe that our findings have important implications for research exploring the effect of contextual variables—such as participants' mood—on complex emotion recognition. For instance, people with a predisposition to experience others' positive emotions may be more often in a positive mood, as suggested by theories of mood-dependency (e.g., Bower, 1991; Gendolla, 2000). According to these theories, affective states serve as contextual cues that increase the availability of thoughts and memories of a similar hedonic tone; they create a preference for exposure to mood-congruent information, and a tendency to process mood-congruent information in greater detail. People in a positive mood have a tendency to recall mainly positive events and situations, to focus their attention on other's positive emotions, and to rate other's emotional expressions as more positive. The opposite is true for people in a negative mood (for reviews, see Matt et al., 1992; Clore et al., 1994; Forgas, 1995), as also confirmed by a wide range of data collected in clinical populations (see Teasdale et al., 1995; Gangemi et al., 2007). It would be interesting to investigate whether directly manipulating participants' mood affects the ability to detect faked enjoyment expressions. If our interpretation of the results is correct, then inducing a positive mood should worsen participant's ability to detect faked enjoyment expressions, while a negative mood should improve smile recognition performance.

Another interesting domain for future investigation is whether other forms of empathy are related to smile authenticity recognition. Empathy is an ability composed by a variety of neurocognitive processes of different complexity and mediated by distinct neural circuits (see Preston and de Waal, 2002; Decety and Lamm, 2006; Singer, 2006). Emotional contagion represents a very basic component of empathy, but at least two other components do exist, namely emotional and cognitive empathy. *Emotional empathy* refers to the cognitive and neural processes that produce a congruent emotion in the observer in response to others' directly perceived emotional displays, or to descriptions of others' emotion-laden experiences (Saxe, 2006). *Cognitive empathy* implies the conscious mental representation of the state of another individual, and it is often conflated with Theory of Mind, i.e., the ability to represent the mental states of others (Premack and Woodruff, 1978). It has been proposed that both emotional and cognitive empathy are related to emotion recognition ability, even if evidence in this respect is not straightforward (see Zaki et al., 2008). Investigating whether people with higher emotional and/or cognitive empathy are better in smile authenticity recognition would add significantly to the present findings, allowing a better characterization of how empathy affects smile authenticity recognition.

Our study has some limitations, which may have lead to underestimate the role of susceptibility to emotional contagion in smile recognition. First, our smile recognition task was based on the manipulation of a single perceptual cue—the Duchenne marker. Recent evidence indicates that symmetry and dynamic features can be even better predictors of participants' ability to assess smile sincerity, especially when dynamic facial displays are evaluated (e.g., Krumhuber and Manstead, 2009). As movement represents a cue aspect in emotion simulation (Goldman and Sripada, 2005), it is plausible to expect that emotional contagion predicts an even greater amount of individual variation in smile recognition when using more naturalistic, dynamic facial expressions. Second, there is evidence that ratings of smile genuineness differ depending on the gender of the encoder (Krumhuber et al., 2007), with females' smiles rated as more faked compared to males' smiles. For this reason, it would be important to replicate these results with smile recognition tasks including male and female faces as stimuli.

### Conflict of interest statement

The authors declare that the research was conducted in the absence of any commercial or financial relationships that could be construed as a potential conflict of interest.
